# Limonin, a natural ERK2 agonist, protects against ischemic acute kidney injury

**DOI:** 10.7150/ijbs.82417

**Published:** 2023-05-29

**Authors:** Xianke Zhou, Yadie Xiang, Dier Li, Menghua Zhong, Xue Hong, Yuan Gui, Wenjian Min, Yudan Chen, Xi Zeng, Haili Zhu, Youhua Liu, Shijia Liu, Peng Yang, Fanfan Hou, Dong Zhou, Haiyan Fu

**Affiliations:** 1 Division of Nephrology, Nanfang Hospital, Southern Medical University; State Key Laboratory of Organ Failure Research; National Clinical Research Center for Kidney Disease; Guangdong Provincial Institute of Nephrology; Guangdong Provincial Key Laboratory of Renal Failure Research, Guangzhou, 510515, China.; 2 Division of Nephrology, Department of Medicine, University of Connecticut School of Medicine, Farmington, CT, 06030, USA.; 3 State Key Laboratory of Natural Medicines and Jiangsu Key Laboratory of Drug Design and Optimization, China Pharmaceutical University, Nanjing, 210009, China.; 4 Department of Clinical Pharmacology, The Affiliated Hospital of Nanjing University of Chinese Medicine, Nanjing, 210029, China.

**Keywords:** limonin, acute kidney injury, extracellular signal-regulated kinase 2, cell death, cell proliferation

## Abstract

Acute kidney injury (AKI) is a refractory clinical syndrome with limited effective treatments. Amid AKI, activation of the extracellular signal-regulated kinase (ERK) cascade plays a critical role in promoting kidney repair and regeneration. However, a mature ERK agonist in treating kidney disease remains lacking. This study identified limonin, a member of the class of compounds known as furanolactones, as a natural ERK2 activator. Employing a multidisciplinary approach, we systemically dissected how limonin mitigates AKI. Compared to vehicles, pretreatment of limonin significantly preserved kidney functions after ischemic AKI. We revealed that ERK2 is a significant protein linked to the limonin's active binding sites through structural analysis. The molecular docking study showed a high binding affinity between limonin and ERK2, which was confirmed by the cellular thermal shift assay and microscale thermophoresis. Mechanistically, we further validated that limonin promoted tubular cell proliferation and reduced cell apoptosis after AKI by activating ERK signaling pathway *in vivo*. *In vitro* and *ex vivo*, blockade of ERK abolished limonin's capacity of preventing tubular cell death under hypoxia stress. Our results indicated that limonin is a novel ERK2 activator with strong translational potential in preventing or mitigating AKI.

## Introduction

An abrupt loss of kidney function characterizes acute kidney injury (AKI) occurring in a few hours or days. AKI is a common but refractory clinical syndrome associated with high morbidity and mortality 1-3. Because of its complicated pathophysiological changes, AKI patients are often prone to severe complications such as fluid disturbances and metabolic acidosis. They subsequently progress to the chronic stage and even enter end-stage renal disease. Physicians need to face the current real scenario of no curative and effective treatment options explicitly targeting AKI in the clinic 4. Therefore, developing effective pharmaceutic agents for AKI treatment is critical to mitigating this medical burden globally.

Several strategies, such as cell or gene therapies, have been evaluated in treating AKI in the past decades 4-6. Unfortunately, none of these approaches have been successfully translated into clinical application 7, 8. In fact, after AKI, the injury/repair process is initiated by circulating factors whose cellular effects are mediated by activated selective signaling pathways. The extracellular signal-regulated kinase (ERK) cascade is a classic target among the diseased kidneys' vast array of activated signals 9-13. Activation of ERK in AKI is renoprotective, although sporadical cases reported opposite findings 14-17. Nevertheless, there are no mature ERK activators for clinical use thus far.

Also known as evodin or obaculactone, limonin is a triterpenoid compound extracted from citrus 18. Under various medical circumstances, it possesses multiple biological effects, including anti-bacterial, anti-inflammatory, anti-oxidant, and anti-proliferative effects 19-22. In particular, limonin exhibited protective effects on organ repair such as heart, brain, and liver after ischemic injury 23-26. These organ-protective effects of limonin undoubtedly lay a theoretical basis for expanding its application to treat kidney disease. However, the efficacy of limonin in dealing with ischemic AKI and the underlying mechanisms remain unclear. In the present study, we identified limonin as a novel ERK2 agonist, and it is a strong candidate for dictating kidney fate after ischemic AKI. Our findings shed light on developing valuable resources to prevent or mitigate AKI in the field.

## Materials and Methods

### Compounds

Limonin (purity > 96.8%) was obtained from the Zhejiang Nanyang Pharmaceutical Group (China). HS15, a new type of amphiphilic solubilizer with high solubilizing capacity and excellent safety, was purchased from Sigma (USA) and used as a solvent in the present study. Limonin was dissolved in distilled water containing 25% HS15.

### Human kidney biopsies samples

Human kidney biopsies specimens were obtained from the Affiliated Hospital of the Nanjing University of Chinese Medicine and Nanfang Hospital, Southern Medical University. All patients included in the presented study have signed the consent forms before they underwent kidney biopsy. The Ethics Committee on Human Subjects approved the studies using human samples at the Affiliated Hospital of the Nanjing University of Chinese Medicine (Document No.2021NL-063-01-08).

### Mouse models

Male C57BL/6 mice (8 - 10 weeks) were purchased from the Vital River Company (Beijing, China). The bilateral kidney ischemia-reperfusion injury (IRI) mouse model was constructed as described previously 27-29. In brief, mice were anesthetized and the bilateral renal pedicles were clipped for 30 min using microaneurysm clamps. During the ischemic period, body temperature was maintained at 37 °C. At 24 h post reperfusion, mice were euthanized, the blood and tissue samples were collected for various analyses.

**Figure 1:**
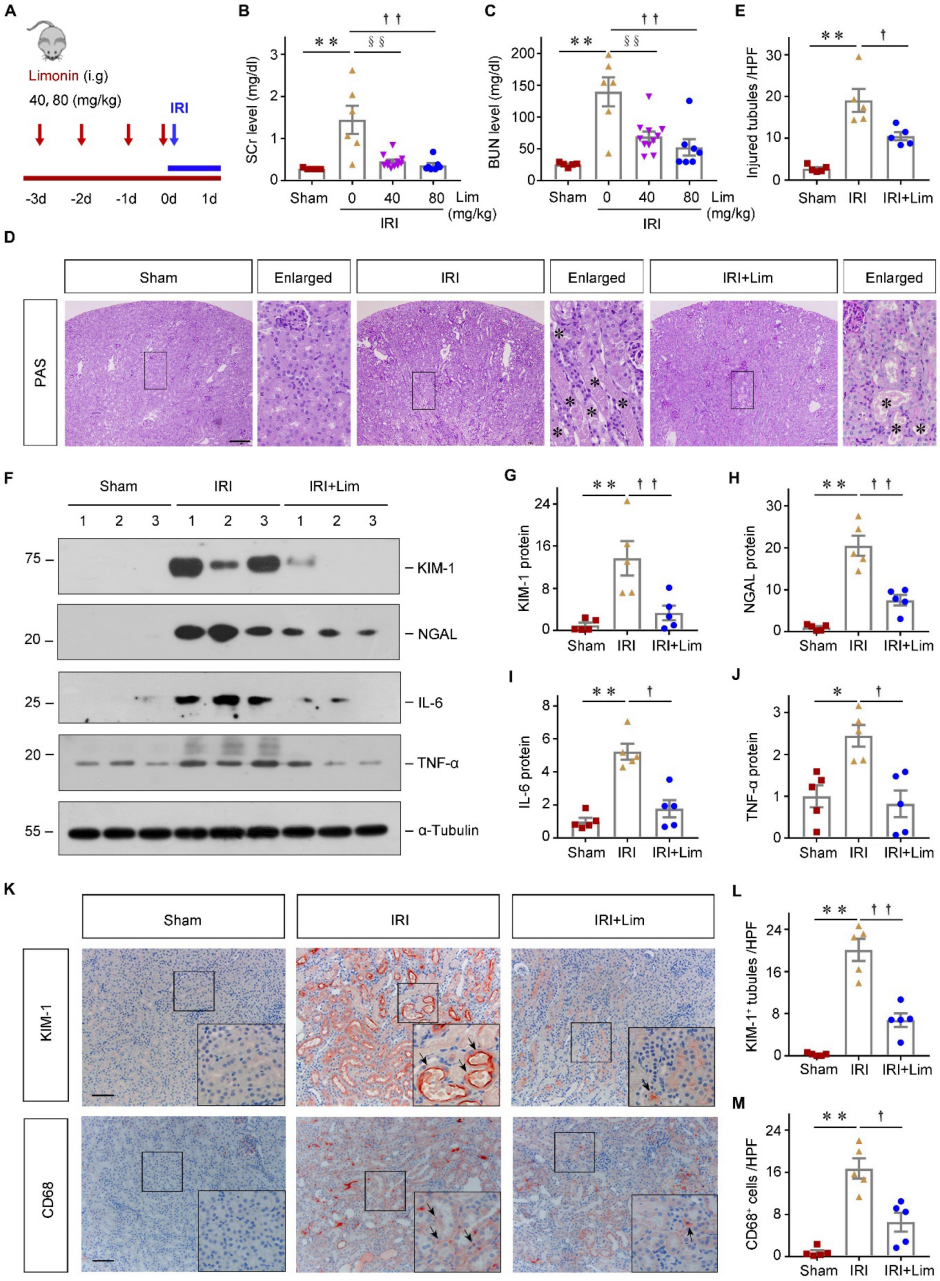
** Limonin-treated mice were resistant to ischemic AKI.** (**A**) Experimental design. The blue arrow indicates the timing of renal IRI surgery. The red arrows indicate the oral gavage of limonin. (**B** and** C**) Serum creatinine (SCr) and blood urea nitrogen (BUN) levels in different groups. (n = 5-11). (**D**) Representative micrographs show kidney injury in different groups. Asterisks in the boxed areas indicate injured tubules. Scale bar, 100 µm. (**E**) Quantitative assessment of kidney injury at 1 day after IRI. Data are presented as numbers of injured tubules *per* high power field (HPF). (n = 5). (**F**) Western blot analyses show KIM-1, NGAL, IL-6 and TNF-α expression in different groups. Quantitative data (**G - J**) are presented. Numbers (1-3) indicate each individual animal in a given group. (n = 5). (**K**) Micrographs show the expression of KIM-1 and CD68**^+^** macrophage infiltration in different groups. Arrows in the boxes indicate positive staining. Scale bar, 100 µm. (**L**) Quantitative data of KIM-1**^+^** tubules. (n = 5). (**M**) Quantitative data of CD68^+^ macrophages. (n = 5). **P < 0.01 versus sham controls; §§*P* < 0.01 versus IRI + limonin (40 mg/kg), ††*P* < 0.01 versus IRI + limonin (80 mg/kg), †*P* < 0.05 versus IRI + limonin (80 mg/kg).

The detailed animal studies designs were presented in Figure 1A, 8A, and 9A. In Figure 1, mice were randomly assigned to 4 groups (n = 5-11/group) as indicated. Three days before IRI, mice were given either 25% HS15 vehicle or Limonin (by oral gavage for 4 consecutive days). In Figure 8, mice were randomly divided into 3 groups (n = 5/group): sham group, IRI group, and IRI administrated with U0126 group. In Figure 9, mice were randomly divided into 4 groups (n = 5/group) and administered by vehicle or limonin in the absence or presence of U0126 (10 mg/kg) (Cell Signaling Technology, Danvers, MA) injection as indicated. In Figure S1A and 1B, mice were randomly divided into 3 groups (n = 5/group), healthy adult mice were orally administered vehicle or limonin for 4 days. In Figure S1C and S1D, mice were randomly divided into 4 groups (n = 5/group), three days before IRI, mice were given either 25% HS15 vehicle or limonin (by oral gavage for 4 consecutive days) or without any treatment. All procedures involving animals in this study were approved by the Animal Ethics Committee at the Nanfang Hospital, Southern Medical University (Document No. L2018243).

### Renal function assessment

The concentrations of blood urea nitrogen (BUN) and serum creatinine (SCr) were measured by an automatic chemistry analyzer (AU480 Chemistry Analyzer, Beckman Coulter, Atlanta, Georgia).

### Kidney histology and immunohistochemical staining

Paraffin-embedded mouse tissue sections were prepared by routine procedures. The heart, liver, lung, and spleen sections were stained with Hematoxylin-Eosin (H.E.), and kidney tissue sections were subjected to Periodic acid-Schiff (PAS) staining. The injured tubules were defined by the presence of tubular dilation, atrophy, intraluminal casts, loss of proximal tubular brush border, and tubular cell vacuolization and detachment. At least 10 random fields were selected for each kidney in the cortex-medulla junctional area and the images were evaluated by three experienced technicians independently. The degree of tubular injury was assessed semi-quantitatively according to the number of injured tubules in the high power field (HPF) by three experienced technicians in a blind manner. Immunohistochemical (IHC) staining was performed according to the established protocol as described previously 28. The antibody information was described in Table S6.

### Cell Culture and Treatment

Human kidney proximal tubular epithelial cells (HK-2) and human embryonic kidney cells (293T) were obtained from the American Type Culture Collection (Manassas, VA, USA) and maintained according to the supplier's recommendations. Primary tubule epithelial cells (PTECs) were isolated from the 8 - 10 weeks male mice kidneys using the established method 30. Briefly, the kidneys were digested by collagenase type IV solution (Invitrogen, USA) for 40 min at 37 °C. Then gradient centrifugation with 32% percoll was used to collect tubules (the pellet). Finally, the cells were seeded on plates in DMEM/F12 with 10% FBS and 1% penicillin-streptomycin.

For *in vitro* study, serum-starved HK-2 (or PTECs) cells were treated with limonin at 20 nM in the absence or presence of U0126 (10 µM) as indicated. The cells were then subjected to western blot analyses and EdU incorporation assay. To mimic IRI *in vivo*, HK-2 cells were placed in a hypoxia incubator chamber (94% N_2_, 5% CO_2_, and 1% O_2_) for 24 h and then in reoxygenation condition (5% CO2, 95% air) for 2 h. Limonin (20 nM) or U0126 (10 µM) was pretreated in the culture medium for 2 h according to different experimental purposes. The cells were harvested for flow cytometry, TUNEL assay, and western blot analyses, respectively.

### Plasmid transfection

The overexpression plasmids of wild type ERK2 (pcDNA3.1-wtERK2-EGFP), mutant ERK2 (pcDNA3.1-mERK2-EGFP) or control plasmid pcDNA3.1-EGFP were purchased from Fenghui Biotechnology (China). 293T cells were transfected with the indicated plasmids separately by Lipofectamine™ 2000 (Invitrogen, Carlsbad, CA, USA) following the manufacturer's instructions. The transfection efficiency was confirmed by fluorescence microscopy and western blot analysis. At 24 h post transfection, 293T cells were collected and prepared for microscale thermophoresis (MST) assay.

### Western blot analysis

Kidney tissues or culture cells were lysed with radioimmune precipitation assay (RIPA) buffer. The supernatants were collected after centrifugation at 13,000 × g at 4 ℃ for 15 min. Protein expression was analyzed by western blot analysis as described previously 31. The antibody information is summarized in Table S6.

### Flow cytometry analysis

The cells were labeled with dyes in PE Annexin V Apoptosis Detection Kit (Becton Dickinson, USA), and then analyzed by flow cytometer (BD FACS Calibur System, Franklin Lakes, NJ, USA). Cells population were classified based on whether it was labeled with neither Annexin V (AV) nor 7-AAD (viable cells), AV alone (apoptotic cells), 7-AAD alone (necrotic cells), or both AV and 7-AAD (late apoptotic cells), as described previously 32.

### TUNEL assay

The terminal deoxynucleotidyl transferase-mediated deoxyuridine triphosphate nick end labeling (TUNEL) staining was performed to detect apoptosis using DeadEnd Fluorometric TUNEL System (Promega, USA). Frozen sections or cell slides were fixed in 4% paraformaldehyde at room temperature. After washing, an incubation mixture containing nucleotide mix and rTdT enzyme was added and incubated for 1 h at 37 °C. Cell nuclei were counterstained with DAPI (C1006, Beyotime) for 15 min. The slides were observed by fluorescence microscope (Olympus, Tokyo, Japan). Apoptosis was expressed as the number of the TUNEL**^+^** cells per HPF.

### EdU labeling and detection

For assessing cell proliferation *in vivo*, 5-ethynyl-2'-deoxyuridine (EdU) (50 mg/kg) (Aladdin, China) was injected into mice by tail vein 4 h before sacrifice. To assess cell proliferation *in vitro*, cells were treated as indicated and then incubated with EdU (10 μM) for 2 h before fixation. To detect the EdU signal, the EdU Click-iT reaction solution (Invitrogen, USA) was performed according to the manufacturer's protocol. The fixed cell slides were incubated with the EdU reaction mixture for 30 min at room temperature. Hoechst reaction solution was then used to stain the cell nuclei. For the kidney sections, co-staining after EdU with an anti-Laminin antibody was performed to characterize the proliferating cells. The stained samples were viewed by fluorescent microscopy (Olympus, Tokyo, Japan).

### Screening potential targets of limonin and identifying AKI-related targets

The structure of limonin was searched in Pubchem's online tool. Subsequently, the structural information was uploaded into PharmMapper Server, a platform for identifying the potential targets based on inverse-docking approaches, to acquire potential targets of limonin 33, 34.

The AKI-associated genes were obtained from the GeneCards database. By searching "acute kidney injury", the obtained genes were identified as the AKI-related targets. All websites used were listed in Table S5.

### Identification of key targets of limonin on AKI

The common targets between limonin and AKI were identified by Funrich software to take the intersection. Then, the Protein-Protein Interaction Networks (PPI) data of these targets was made by the String database 35, and downloaded PPI data was visualized by the Cytoscape software 36. Subsequently, the important nodes in the PPI network were obtained with plug-in CytoNCA of Cytoscape. Based on the centrality values (degree centrality, betweenness centrality, and closeness centrality), the top 10 ranked nodes were chosen as the key targets of limonin in AKI. All websites used were listed in Table S5.

### Molecular docking of limonin to ERK2

The crystal structure of ERK2 was acquired from Protein Data Bank (PDB) database (PDB ID: 4FV3), and the 3D molecular structure of limonin was sketched by PubChem (PubChem CID:179651). Structures of ERK2 and limonin were prepared for docking using the Discovery Studio.

### Microscale thermophoresis (MST)

MST assay was applied to determine the binding affinity of ligand (limonin) and target (ERK2). First, the fluorescently labeled plasmid (wtERK2-EGFP, mERK2-EGFP or free EGFP as control) was transfected into 293T cells. After stable expression, the cell lysates were extracted from 293T cells, respectively. Then, the lysates were diluted in PBS-T buffer (containing 0.05 (v/v) % Tween-20, pH 7.2) to a final concentration at which the fluorescent signals of the EGFP proteins were well above the detection limit of the Monolith NT.115 instrument (NanoTemper). Limonin (1 mM) was serially diluted in a 1:1 ratio into 16 gradient concentrations. The binding reaction systems of wtERK2-EGFP-limonin, mERK2-EGFP-limonin and EGFP-limonin were incubated for 30 min at room temperature and then loaded into NT.115 standard coated capillaries (NanoTemper). MST measurements were performed on Monolith NT.115 instrument at 40% MST power and 20% LED Power. Dose-response curves (at least 12 concentration points) for the binding interactions between limonin and ERK2 were fitted to calculate the Kd value.

### Cellular thermal shift assay (CETSA)

The thermal shift assay was performed according to the previously described protocol with modification 37-39. For the temperature-dependent thermal shift assay, HK-2 cells were seeded equally in two 10 cm dishes and allowed to reach 80% confluence. One day later, cells in each dish were incubated with 20 μM limonin or an equal volume of DMSO at 37 °C for 1 h. After trypsinization and washing with PBS, cells were resuspended in 1.3 mL PBS containing freshly added protease inhibitors and were divided evenly among 12 tubes. Cells in each tube were heated at the indicated temperatures for 3 min and kept at room temperature for 3 min. Heated cells were lysed by three cycles of freezing in liquid nitrogen for 1 min and thawing in water at room temperature for 1 min. The cell lysates were centrifuged at 20,000 × g for 20 min at 4 °C. The soluble fractions were isolated for Western blotting analysis.

For the dose-dependent thermal shift assay, HK-2 were lysed and divided evenly among 8 tubes (50 µL/tube). After incubation with various concentrations of limonin (between 0 to 100 µM), the lysates were heated at 52 °C for 3 min. Then, the supernatant was isolated and subjected to immunoblotting analysis of ERK2 as described above.

### Statistical analysis

The statistical analysis was carried out with SPSS 23.0 (SPSS Inc, Chicago, IL) or GraphPad Prism 7.0 (GraphPad Software, USA). The data normality and homogeneity of variance were checked by Kolmogorov-Smirnov test and Levene's test, respectively. All data were presented as mean ± SEM. The Tm_50_ value was obtained by probit analysis. For comparison of two groups, Student's t-test was conducted. For comparison of more than two groups, one-way ANOVA followed by Dunnett's T3 test or LSD multiple comparisons test were used. *P* values <0.05 are considered statistically significant.

## Results

### Limonin mitigates ischemic AKI

To evaluate the efficacy of limonin in AKI, mice were orally administered limonin 3 days before IRI surgery (Figure 1A). At 24 hours after IRI, we first assessed the kidney function changes. Serum creatinine and blood urea nitrogen levels were significantly decreased in the limonin treatment group compared to the vehicle group (Figure 1, B-C). The optimized dose (80 mg/kg) was chosen for all subsequent *in vivo* experiments 21, 40. Consistently, PAS staining revealed less severe morphological damages in limonin-treated mice after ischemic AKI (Figure 1, D-E). Western blot analyses revealed that the inductions of kidney injury molecule-1 (KIM-1) and neutrophil gelatinase-associated lipocalin (NGAL), the two classic markers reflecting AKI severity, were markedly reduced in limonin-treated kidneys compared to vehicles (Figure 1, F-H). Immunohistochemical staining consistently confirmed that KIM-1 induction was markedly reduced in kidney tubular cells after being treated with limonin, compared to vehicles (Figure 1, K-L).

Because inflammation is a key pathological factor in AKI development and progression, we then determined whether limonin influences the kidney inflammatory status after ischemic AKI. As shown in Figure 1F, western blot analyses revealed that limonin reduced the levels of proinflammatory cytokines such as IL-6 and TNF-α in the diseased kidneys compared to vehicles (Figure 1, I-J). Consistently, limonin remarkably repressed the infiltration of CD68^+^ macrophages in the diseased kidneys after ischemic AKI (Figure 1, K-M).

Limonin is a natural monomer compound extracted from citrus. To assess its systemic toxicities *in vivo*, both healthy adult and ischemic AKI mice were orally administered with limonin and there are no apparent differences in survival or behavior of the mice between solvent and limonin treatment. Then, the mice were sacrificed on day 5, blood and vital organs were harvested. Compared with vehicle group, blood biochemistry analysis indicated that limonin treatment did not damage the liver and kidney function, as presented in Table S1. HE staining demonstrated no histological injury to the heart, lung, liver, spleen, and kidney in this groups (Figure S1, A-B). In addition, the side effects were further assessed in ischemia AKI mice. As shown in Figure S1C and S1D, no obvious histological difference was observed among the different groups. Western blot analysis confirmed the kidney not suffers injury after limonin treatment (Figure S1, E and H). Taken together, these results suggested that, with favorable safety profiles, limonin exhibits therapeutic potential in mitigating ischemic AKI.

### Limonin represses tubular cells apoptosis and promotes their proliferation after AKI

**Figure 2:**
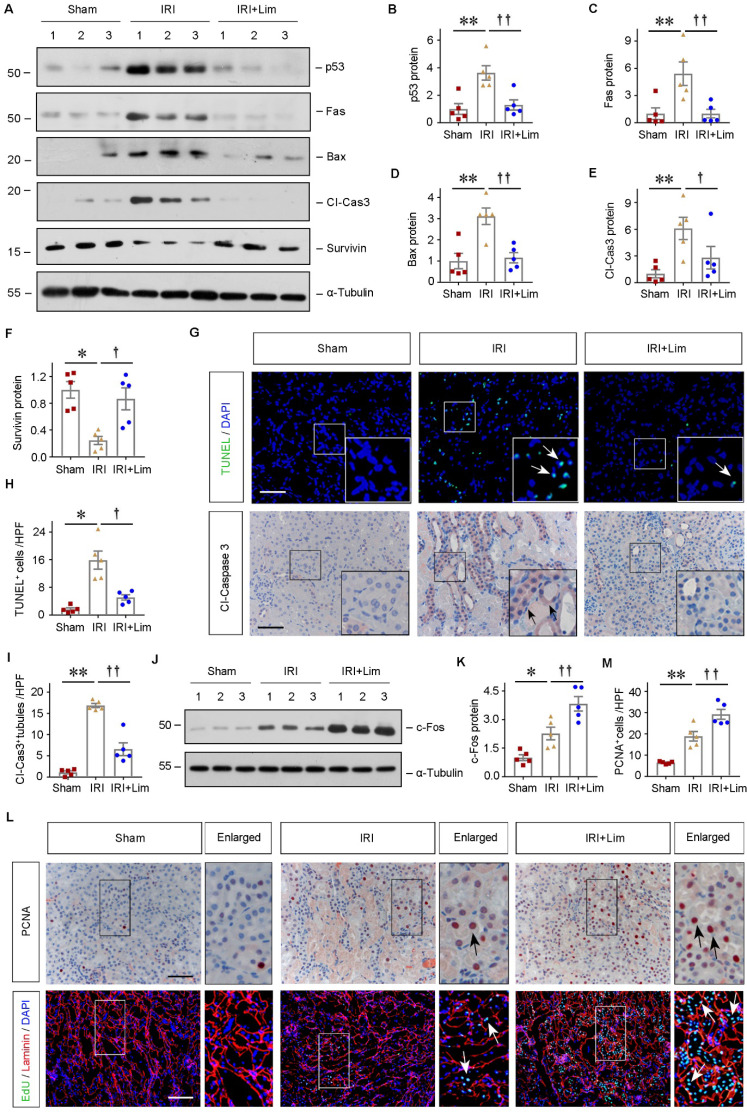
** Limonin inhibits tubular cells apoptosis and promotes their proliferation.** (**A - F**) Representative western blots (A) and quantitative data of p53 (B), Fas (C), Bax (D), cleaved caspase3 (E) and Survivin (F) are shown. (n = 5). (**G**) Representative micrographs show that apoptotic cells detected by TUNEL staining and immunostaining for cleaved caspase3. Arrows indicate apoptotic cells. Scale bar, 100 µm. Quantitative determination of TUNEL**^+^** cells (**H**) and cleaved caspase3^+^ tubules (**I**) in different groups. (n = 5). (**J - K**) Representative Western blots (J) and quantitative data of c-Fos (K) are shown. (n = 5). (**L**) Cell proliferation was assessed by immunostaining for PCNA (upper panels) or co-immunostaining of EdU (green), basement membrane protein laminin (red) and nuclei (blue) (lower panels). Scale bar, 100 µm. (**M**) Quantitative determination of PCNA^+^ cells in different groups. (n = 5). **P < 0.01 versus sham controls; ††*P* < 0.01 versus IRI + limonin (80 mg/kg); †*P* < 0.05 versus IRI + limonin (80 mg/kg).

To explore the mechanisms underlying the renoprotection of limonin against AKI, we examined cell apoptosis and proliferation in the kidneys after various treatments. As shown in Figure 2A-2F, the expression of pro-apoptosis proteins, including p53, Fas, Bax, and cleaved caspase3, were significantly induced in the ischemic kidneys, which were largely repressed by limonin. Conversely, as a member of the apoptosis family inhibitor, the survivin expression was markedly upregulated after limonin treatment. To understand the distributions of apoptotic cells in the diseased kidneys after ischemic AKI, we employed TUNEL staining approach. As shown in Figure 2G and 2H, increased TUNEL**^+^** cells were observed in the ischemic kidneys, while limonin significantly reduced the numbers of apoptotic cells. IHC staining for cleaved caspase3 further revealed that most apoptotic cells were renal tubular cells after ischemic AKI, and cleaved caspase3^+^ cells were reduced after limonin treatment (Figure 2G and 2I). After AKI, tubular cell death and proliferation balance directly determine short- and long-term kidney outcomes. Therefore, c-Fos, the proliferation-associated protein, was examined by western blot assay. As shown in Figure 2J and K, limonin promoted tubular cell proliferation after IRI compared to vehicles. IHC staining for PCNA confirmed that limonin promoted tubular cell proliferation (Figure 2, L-M). To further corroborate this finding, we performed the EdU assay, a sensitive assay to measure cell proliferation by detecting and quantifying DNA synthesis. Compared to vehicles, limonin significantly increased EdU**^+^** cells in the diseased kidneys after ischemic AKI (Figure 2L). These proliferating cells appeared to be tubular cells, as shown by co-immunostaining with laminin. Our results suggested that limonin mitigates AKI by repressing tubular cell apoptosis and promoting cell proliferation after IRI.

### Limonin protects tubular cells against apoptosis and promotes their proliferation *in vitro*

**Figure 3:**
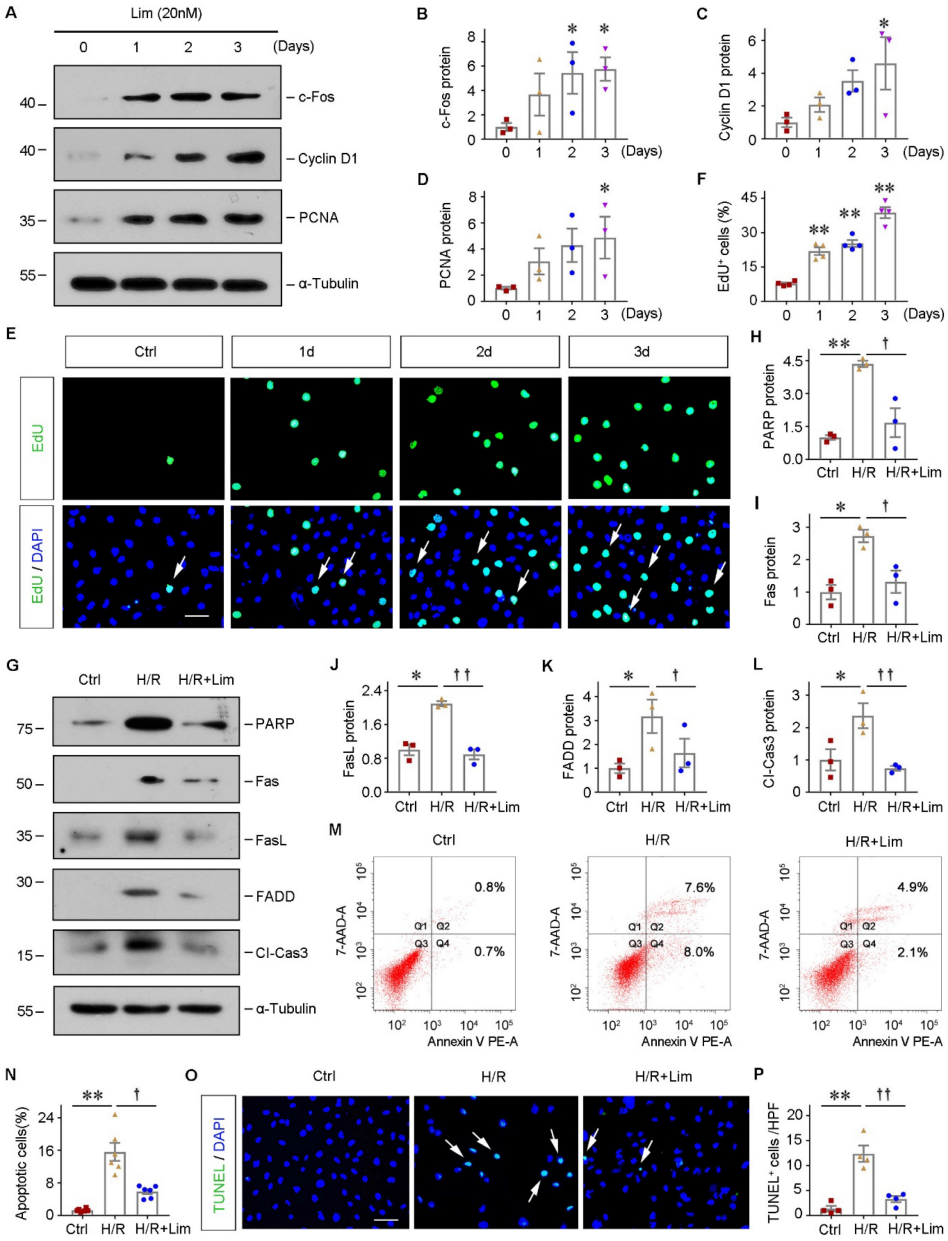
** Limonin protects tubular cells against apoptosis and promotes their proliferation* in vitro.*** HK-2 cells were incubated with limonin (20 nM) as indicated time. (**A** - **D**) Representative western blots (A) and quantitative data (B - D) show c-Fos, Cyclin D1, and PCNA levels in different groups. (n = 3). (**E** and **F**) EdU incorporation assay shows that limonin promoted tubular cell DNA synthesis. Representative EdU incorporation assay (E) and quantitative data (F) are shown. Scale bar, 50 µm. (n = 4). (**G** -** L**) limonin protects tubular cells against apoptosis induced by hypoxia/reoxygenation (H/R). HK-2 cells were pretreated with limonin (20 nM) for 2 h, then incubated in hypoxic condition (1% O_2_) for 24 h, and then reoxygenation for 2 h. Western blot (G) and quantitative data (H - L) show protein expression of PARP, Fas, FasL, FADD, and cleaved caspase3 in various groups. (n = 3). (**M** and** N**) Flow cytometry shows that limonin reduced H/R-induced cell apoptosis. Representative histograms (M) and quantitative data (N) are shown. (n = 6). (**O** and **P**) TUNEL staining (O) shows that limonin reduced H/R-induced cell apoptosis, and quantitative data (P) are presented. Scale bar, 50 µm. (n = 4). *P < 0.05, **P < 0.01 versus controls; ††P < 0.01, †*P* < 0.05 versus H/R.

Next, the role of limonin in regulating tubular cells growth and survival was investigated *in vitro*. As indicated, we incubated HK-2 cells with limonin for various periods. As shown in Figure 3A-3D, limonin promoted multiple proliferation-related proteins expression, including c-Fos, Cyclin D1, and PCNA, in a time-dependent manner. The EdU incorporation assay obtained similar results. As shown in Figure 3E and F, increased EdU incorporation was observed in HK-2 cells after incubation with limonin compared to controls. These results demonstrated that limonin promoted kidney tubular epithelial cell proliferation *in vitro*.

To further mimic IRI *in vivo*, HK-2 cells were subjected to hypoxia/reoxygenation (H/R) injury in the presence or absence of limonin. As shown in Figure 3G-3L, H/R induced the expression of various apoptosis-related proteins, including poly (ADP-ribose) polymeras [PARP], Fas, Fas ligand (FasL), Fas-associated death domain (FADD), and cleaved caspase3, and their inductions were significantly abolished by limonin. In addition, through flow cytometry assay, we confirmed that incubation with limonin significantly reduced H/R-mediated tubular cell apoptosis (Figure 3, M-N). TUNEL staining showed similar results (Figure 3, O-P). These results suggest that limonin promotes tubular epithelial cell proliferation and reduces cell apoptosis *in vitro* under hypoxia stress.

### ERK2 is a direct target of limonin

**Figure 4:**
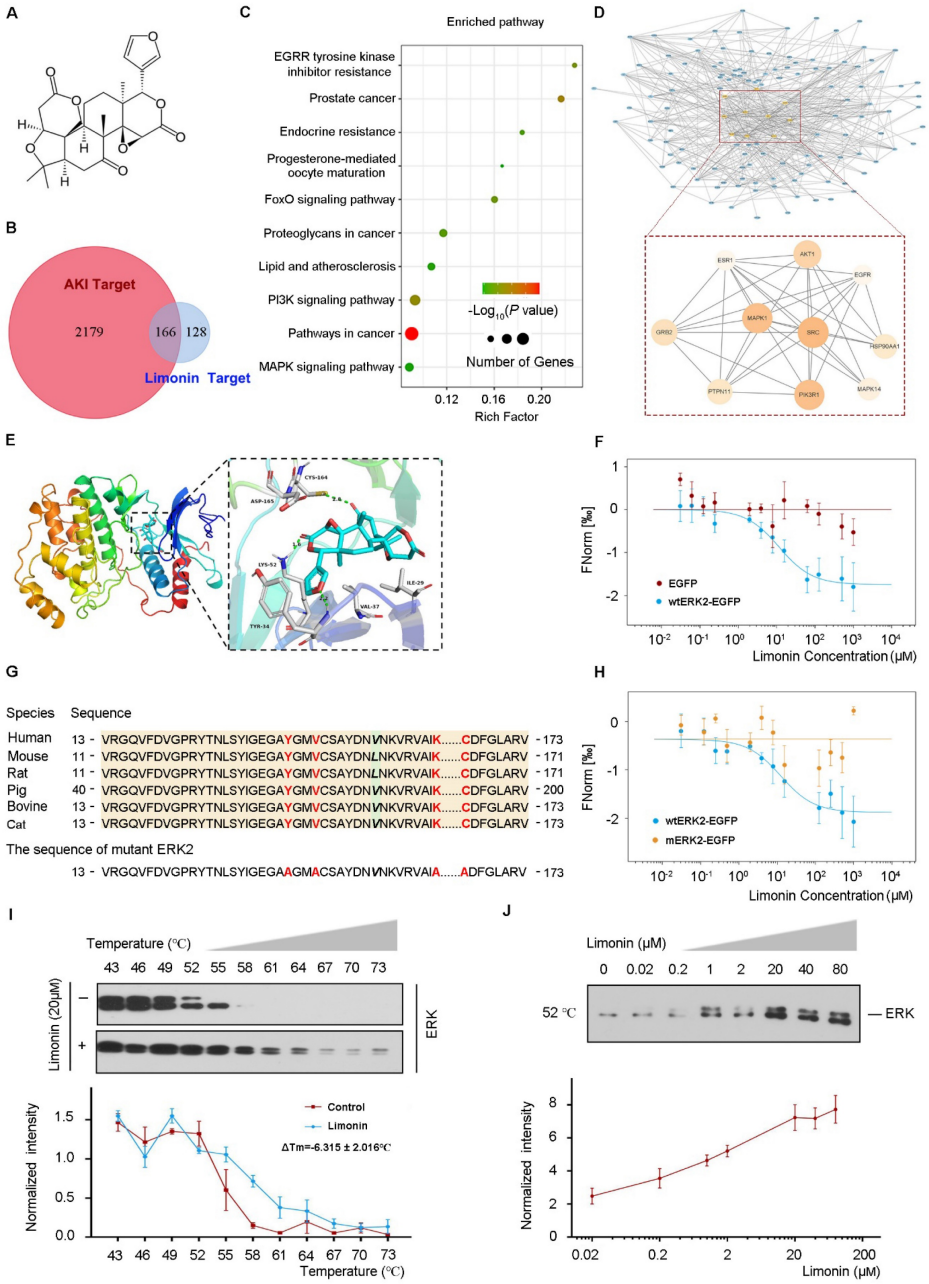
** ERK2 is a direct target of limonin. (A**) 2-D Structure of limonin. (**B**) Venn diagram shows the limonin and AKI-related targets. (**C**) KEGG enrichment for shared target proteins. The size of the bubble indicates the number of genetic factors. The bigger the bubble, the richer the path genes, and the redder the color indicates the pathway enrichment. (**D**) The PPI network of 166 common targets between AKI-related and limonin (upper panel). Zoom-in view of the PPI network of 10 Hub targets (lower panel). (**E**) The 3-D binding model between ERK and limonin. The green dotted lines represent the hydrogen bond. (**F**) MST analysis shows limonin binds to wtERK2-EGFP protein (blue), whereas the empty vector did not generate a binding curve (red). (**G**) Multiple-alignment analysis of human ERK2 and other species showed conservation of four sites. The mutant ERK2 was made by substituting Y34, V37, K52, and C164 residues (red font) with alanine. (**H**) MST analysis shows limonin binds to wtERK2-EGFP (blue) but not the mutant ERK2 (yellow). (**I - J**) limonin (20 μM) increases the thermal stability of ERK detected by the temperature-dependent cellular thermal shift assay (I) and the concentration-dependent cellular thermal shift assay at 52 °C (J). (n = 3).

To delineate the molecular mechanisms of how limonin protects against ischemic AKI, we employed the network pharmacology approach to identify the key targets that are responsible for renoprotection. The summary and description of the study workflow regarding the network pharmacology research and target validation were presented in Figure S2. The 2-D and 3-D structure of limonin were displayed in Figure 4A and Figure S3A, respectively. A total of 294 corresponding limonin targets were obtained from PharmMapper and the top 10 best-fitted targets were listed in Table S2. Through searching in the GeneCards database, 2345 AKI-related targets were identified. Among them, 166 shared protein targets were recognized as probable therapeutic targets, as illustrated in Figure 4B. Kyoto Encyclopedia of Gene and Genomes (KEGG) enrichment analysis of these common targets revealed that several enriched signaling pathways, including MAPK, PI3K, FoxO, and other signaling pathways (Figure 4C).

To identify the core targets of limonin in treating AKI, we constructed the limonin-targets-AKI network by Cytoscape. Based on the degree values (The higher the degree value of the node is, the more critical node in the network), the top 10 targets were selected as core target proteins. The detailed information was shown in Table S3. The PPI network of the core targets included 10 nodes and 34 interaction edges, with an average degree value of 27.4 (Figure 4D). Among these core targets, mitogen-activated protein kinase 1 (MAPK1), also known as ERK2, was structurally best matched with limonin with a fit score of 3. Therefore, we chose ERK2 as a key target for subsequent experimental validation.

To validate the binding of limonin and ERK2, we first performed the molecular docking study. As shown in Figure 4E and Figure S3B - C, tyrosine (Tyr 34), lysine (Lys 52), and cysteine (Cys 164) in ERK2 formed hydrogen bonds with limonin by which stabilized the position of limonin in the ERK2 binding pocket. Then, biophysical approaches were employed to confirm the interactions between limonin and ERK2. Microscale thermophoresis was applied to determine the binding affinity of limonin and ERK2. Limonin exhibited a high affinity to wtERK2-EGFP but not to EGFP control (Figure 4F). The dissociation constant of limonin binding to ERK2 was calculated as 11.56 ± 2.93 μM (Kd), which indicated a strong interaction between them. For further confirmation, we designed ERK2 mutation at the complex interface (Y34, V37, K52, C164) that was predicted to preserve the structural integrity of ERK2 but disrupt the binding of limonin. The four sites were evolutionarily conserved (Figure 4G). As expected, only wild-type ERK2, but not the mutant, exhibited significant binding with limonin (Figure 4H). Similar results were obtained when the physical interaction of limonin with ERK2 was assessed by cellular thermal shift assay. As shown in Figure 4I and J, both temperature- and dose-dependent CETSA data showed that limonin enhanced the thermal stability of ERK but not the α-Tubulin control (Figure S3D - E). The Tm50 values of ERK and α-Tubulin were listed in Table S4. Together, these results clearly indicated that ERK2 is a direct target of limonin.

### Limonin-mediated tubular cells proliferation depends on ERK phosphorylation *in vitro*

**Figure 5:**
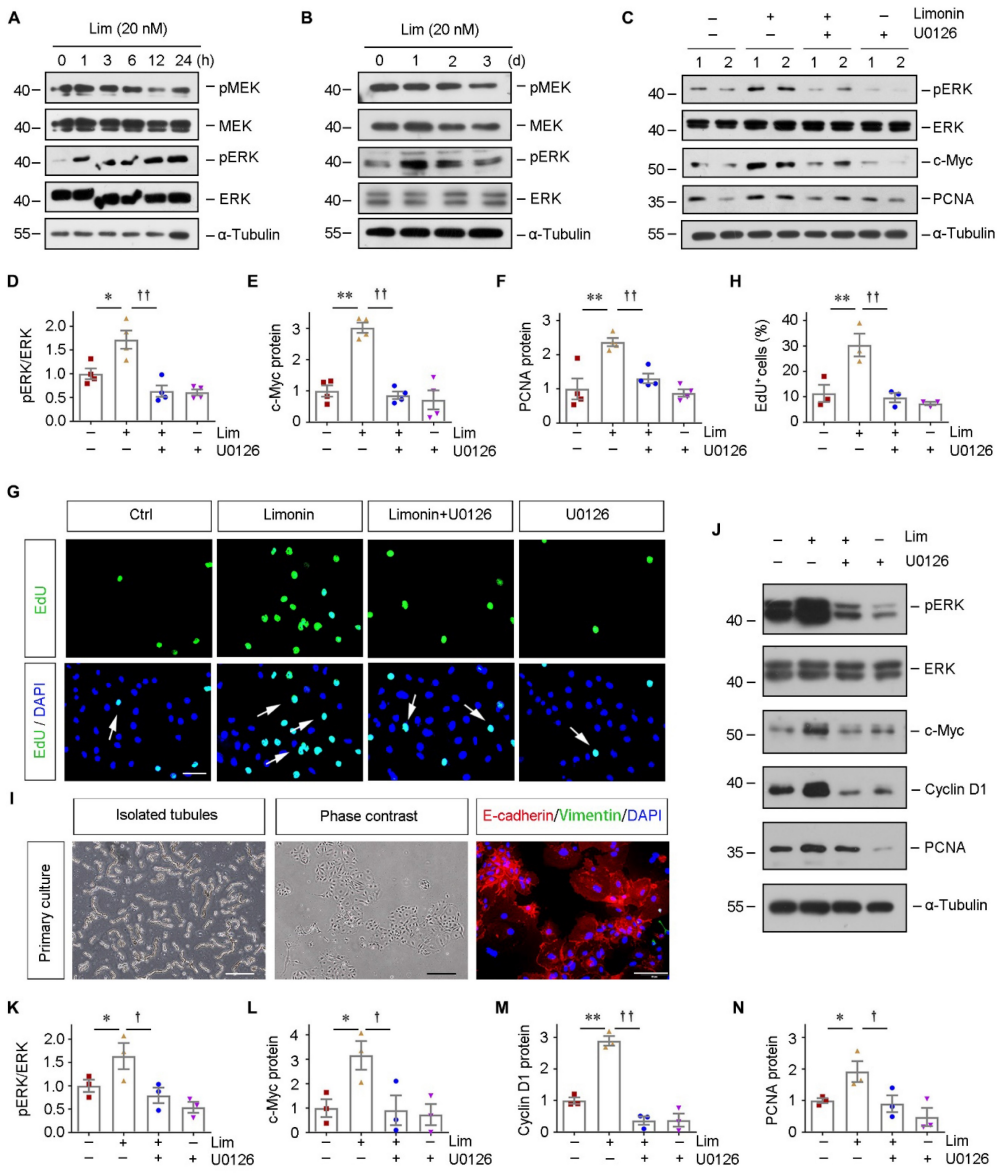
** Limonin-mediated tubular cell proliferation depends on ERK phosphorylation *in vitro.*** (**A** and **B**) HK-2 cells were treated with limonin (20 nM) for various periods of time as indicated. Representative Western blots show the levels of pMEK and pERK after short-term and long-term incubation. (**C - F**) HK-2 cells were treated with limonin (20 nM) alone or co-treated with U0126 (10 μM) for 24 h, and then cell lysates were subjected to western blot analyses for pERK, ERK, c-Myc, and PCNA. Representative Western blots (C) and quantitative data (D - F) are presented. (**G** and **H)** Representative EdU incorporation assay (G) and quantitative data (H) are shown. Arrows indicate proliferative cells. Scale bar, 50 µm. (n = 4). (**I**) Freshly isolated tubules and primary tubular cells are shown (left and middle panels). Scale bar, 500 µm. Primary tubular cells were co-immunostained with antibodies against E-cadherin (red) and vimentin (green) (right panel). Scale bar, 50 µm. (**J**) Representative western blots show protein expression of pERK, ERK, c-Myc, Cyclin D1, and PCNA after various treatments in mouse primary cultured tubular epithelial cells. (**K - N**) Quantitative data. (n = 3). *P < 0.05, **P < 0.01 versus controls; †*P* < 0.05, ††*P* < 0.01 versus limonin group.

Since we identified that ERK2 is a direct target of limonin, we then examined how limonin regulates ERK2. As shown in Figure 5A and 5B, limonin rapidly induced ERK phosphorylation at 1 hour in serum-starved HK-2 cells, whereas the total ERK did not alter. The phosphorylated level of ERK peaked at 24 h and then returned to baseline. By contrast, limonin showed no detectable influences on mitogen-activated protein kinase kinase (MEK), an upstream kinase of ERK. The solvent (DMSO) treatment results were shown in Figure S4A and S4B. Intriguingly, we found that ERK activation seemed to be required for initiating the proliferative response of HK-2 cells to limonin. Blockade of ERK activation by U0126, a specific inhibitor of MEK 41, 42, abolished limonin-induced c-Myc and PCNA expression in HK-2 cells (Figure 5, C-F). EdU incorporation assay consistently demonstrated that blockade of ERK activation abolished limonin-mediated tubular cells proliferation (Figure 5, G-H).

Furthermore, we observed similar results in the employed primary tubular epithelial cells (PTECs) *ex vivo*. Isolated mouse kidney PTECs were identified by immunostaining with specific antibodies against E-cadherin and vimentin (Figure 5I). The cells were then incubated with limonin with or without U0126. As shown in Figure 5J-5N, blockade of ERK activation by U0126 abolished limonin-induced c-Myc, Cyclin D1, and PCNA expression in primary tubular epithelial cells. Collectively, our results indicated that limonin is a direct ERK2 activator that influences tubular cell proliferation.

### Limonin-mediated protection of tubular cells against apoptosis depends on ERK phosphorylation *in vitro*

**Figure 6:**
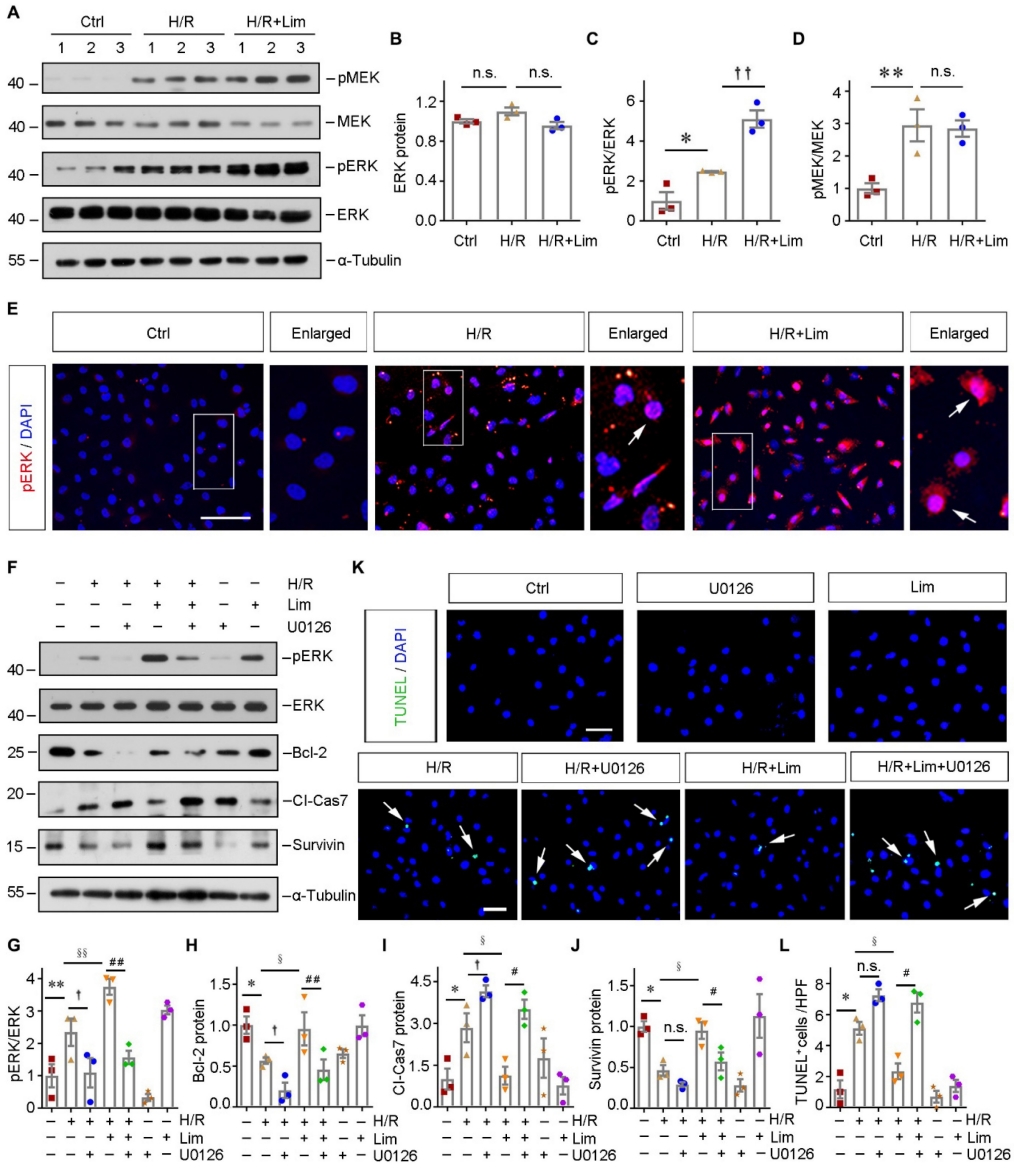
** Limonin-mediated protection of tubular cells against apoptosis depends on ERK phosphorylation *in vitro.*** (**A**) Representative western blots show renal expression of pMEK, total MEK, pERK, and total ERK in different groups as indicated. Numbers (1-3) indicate each individual wells of cells. (**B - D**) Quantitative data are presented. **P < 0.01 versus controls; †*P* < 0.05 versus H/R, (n = 3). (**E**) Immunofluorescence staining of pERK in different group as indicated. Scale bar, 50 µm. Arrows indicate the nuclear translocation of ERK. (**F**) Representative Western blots show protein expression of pERK, total ERK, Bcl-2, cleaved caspase7 and Survivin after various treatments in HK-2 cells. (**G - J**) Graphic presentations are presented. *P < 0.05, **P < 0.01 versus control cells; † (or §) *P* < 0.05, §§*P* < 0.01 versus H/R; #*P* < 0.05, ##*P* < 0.01 versus H/R + limonin, (n = 3). (**K** and **L**) Representative micrographs of TUNEL staining (K) and quantitative data (L) are shown. Arrows indicate TUNEL-positive apoptotic cells. Scale bar, 50 µm. *P < 0.05 versus control cells; † (or §) *P* < 0.05 versus H/R; #*P* < 0.05 versus H/R + limonin. (n = 3).

Besides cell proliferation, we next assessed the effect of limonin on regulating ERK under pathological conditions. As shown in Figure 6A-6D, H/R induced the phosphorylation of MEK and its downstream kinase, ERK. Impressively, the phosphorylation level of ERK was markedly enhanced by limonin without affecting MEK. Immunofluorescence staining revealed that pERK was triggered in both cytoplasm and nuclei of HK-2 cells after H/R, which was further augmented after incubation with limonin (Figure 6E).

As we mentioned in Figure 3, H/R induced apoptosis in HK-2 cells, which was prevented by limonin. However, inhibiting ERK activity by U0126 reduced the expression of anti-apoptosis proteins, including Bcl-2 and survivin, and subsequently restored the level of cleaved caspase7 (Figure 6, F-J). In addition, TUNEL staining consistently demonstrated that limonin prevents tubular cell apoptosis by activating ERK (Figure 6, K-L). These results suggested that limonin reduces tubular apoptosis through phosphorylating ERK.

### Limonin phosphorylated ERK to protect against ischemic AKI

**Figure 7:**
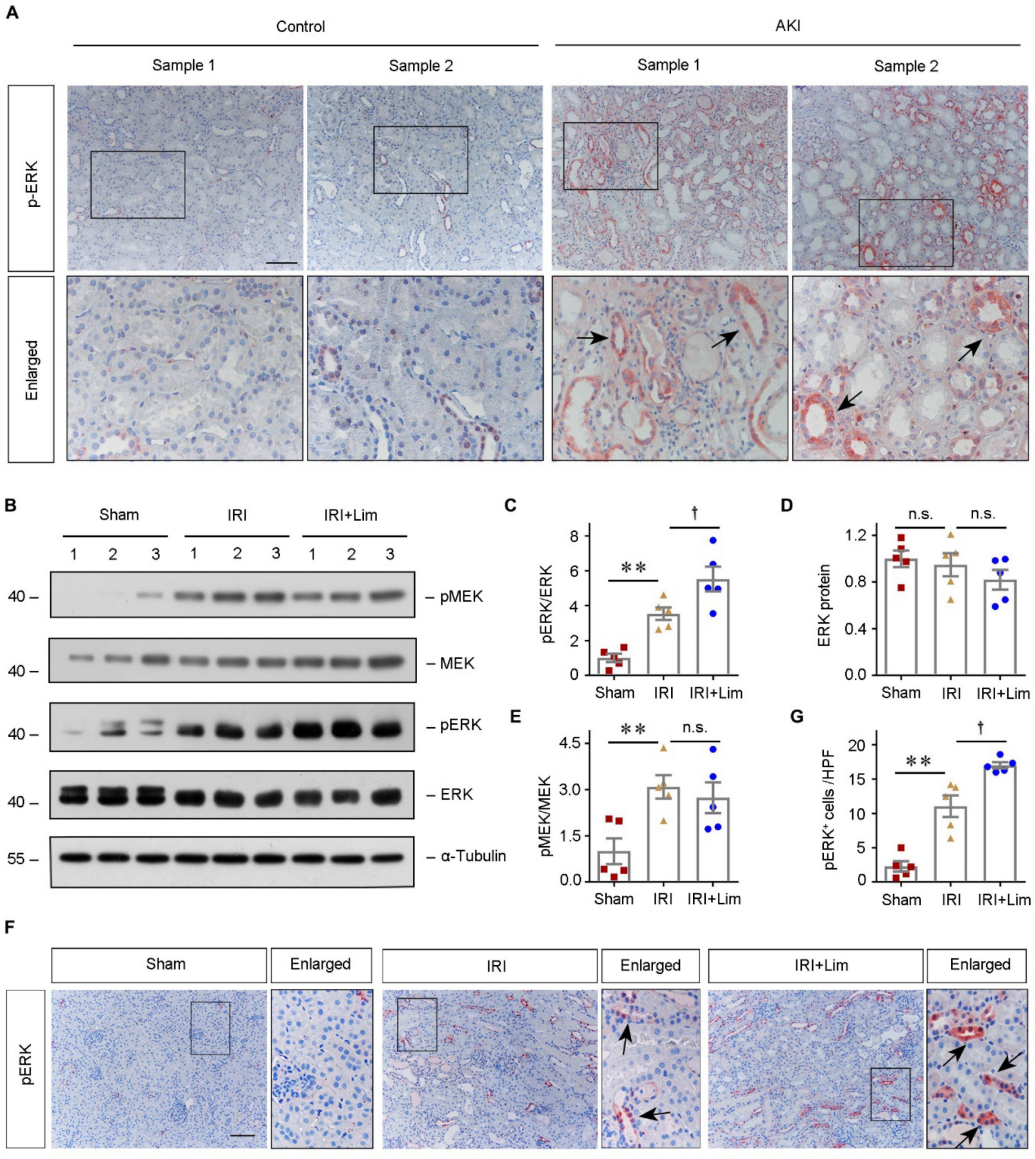
** ERK phosphorylation is a common response to AKI in humans and mice.** (**A**) Representative micrographs show the abundance and localization of pERK proteins in healthy control and AKI patients as indicated. Scale bar, 100 µm. (**B - E**) Western blot analyses of renal pMEK, MEK, pERK and ERK protein expression after IRI in different groups as indicated. Representative western blot (B) and quantitative data (C - E) are shown. Numbers (1-3) indicate each individual animal in a given group. *P <0.05, **P <0.01 versus sham; †*P* <0.05 versus IRI + limonin (80 mg/kg). (n = 5). (**F** and **G**) Representative immunohistochemical staining (F) and quantitative data (G) show renal pERK expression in various groups as indicated. Arrows in the enlarged boxed areas indicate the pERK positive tubules. (n = 5). Scale bar, 100 µm. **P <0.01 versus sham; †*P* < 0.05 versus IRI + limonin (80 mg/kg).

To further evaluate the clinical relevance of ERK phosphorylation in human AKI, we examined the expression of pERK in the kidney biopsy specimens obtained from AKI patients. IHC staining revealed that pERK was dramatically upregulated and predominantly localized in the diseased tubular cells (Figure 7A). Similarly, in the ischemic AKI mouse model, western blot assay indicated significant activation of ERK and its upstream MEK in kidneys after IRI, while limonin pretreatment further phosphorylated ERK (Figure 7, B-E). IHC staining revealed consistent findings on the levels of pERK in the ischemic kidneys (Figure 7, F-G).

**Figure 8:**
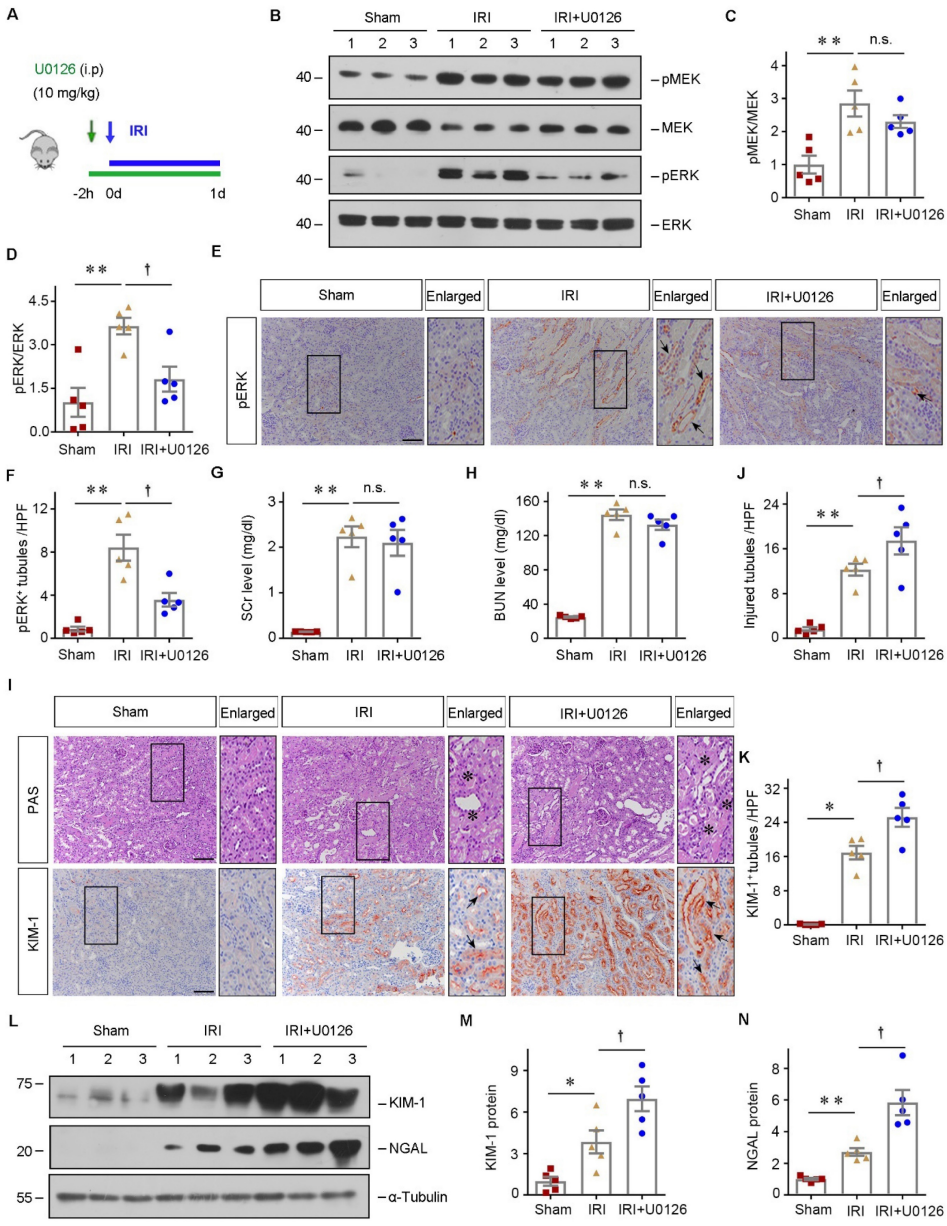
** Pharmacologic inhibition of ERK signaling aggravates kidney injury.** (**A**) Experimental design. The blue arrows indicate the timing of renal IRI surgery. The green arrow indicates the timing of injecting U0126. (**B** - **D**) Western blot analyses show different groups' pMEK, MEK, pERK, and ERK expression. Representative western blot (B) and quantitative data (C - D) are presented. Numbers (1-3) indicate each individual animal in a given group. (n = 5). Representative immunohistochemical staining (**E**) and quantitative data (**F**) show renal pERK expression in various groups. Arrows in the enlarged boxed areas indicate the pERK^+^ tubules. Scale bar, 100 µm. (n = 5). (**G** and **H**) SCr and BUN levels in different groups. (n = 5). (**I**) Micrographs show kidney morphology and KIM-1 expression in different groups. Asterisks or arrows in the enlarged boxed areas indicate injured tubules. Scale bar, 100 µm. (**J** and **K**) Quantitative data are presented. (n = 5). (**L** - **N**) Western blot analyses show KIM-1 and NGAL expression in different groups. Representative western blot (L) and quantitative data (M - N) are presented. Numbers (1-3) indicate each individual animal in a given group. (n = 5). *P < 0.05, **P < 0.01 versus sham controls; †P < 0.05 versus IRI + U0126.

To confirm the role of ERK phosphorylation in mediating the pathogenesis of AKI, we then inhibited ERK using U0126 *in vivo*. The detailed experimental design was shown in Figure 8A. Western blot assay showed that ERK phosphorylation was decreased after U0126 administration (Figure 8, B-D). IHC staining demonstrated that U0126 repressed ERK phosphorylation in the tubules after ischemic AKI, compared to vehicles (Figure 8, E-F). Then we assessed the effects of ERK inhibition on renal function after IRI. SCr and BUN levels were significantly increased in both IRI and IRI+U0126 groups compared with the sham group (Figure 8, G-H). Although there was no difference in kidney function change between IRI and IRI+U0126 group, inhibiting ERK further aggravated the kidney injury (Figure 8, I-J). Immunostaining or western blot assay demonstrated that KIM-1 and NGAL were upregulated in the diseased kidneys after U0126 treatment compared with vehicle (Figure 8, I and K-N). Therefore, our data suggested that inhibiting ERK aggravates kidney damage after ischemic AKI.

### The renoprotective capacity of limonin is abolished after inhibiting ERK in AKI

**Figure 9:**
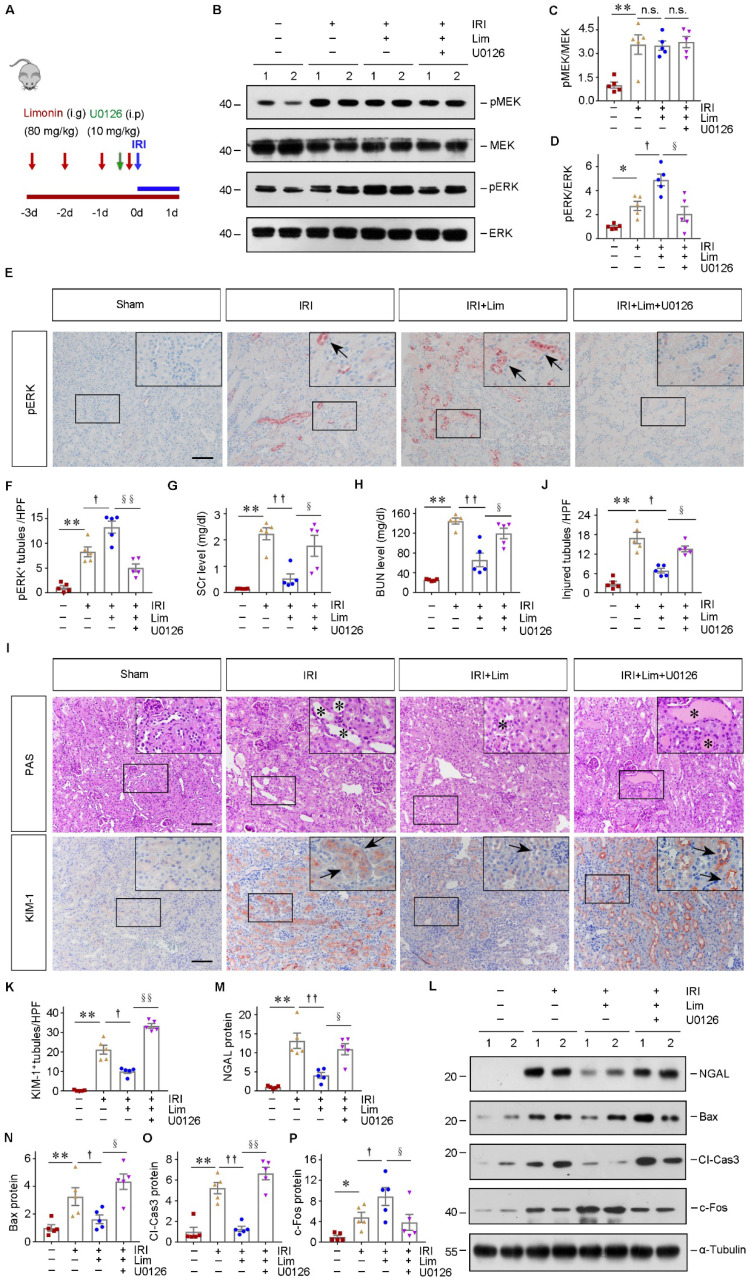
** Pharmacologic inhibition of ERK abolishes renal protection of limonin after AKI.** (**A**) Experimental design. The blue arrow indicates the timing of renal IRI surgery. The red arrows indicate the oral gavage of limonin. The green arrow indicates U0126 injection time. (**B**) Western blots show pMEK, MEK, pERK, and ERK levels in different groups. Numbers (1, 2) indicate each individual animal in a given group. (**C** and **D**) Quantitative data of pMEK and pERK. (n = 5). (**E** and **F**) Immunohistochemical staining (E) and quantitative data (F) show pERK expression in various groups. Arrows indicate the pERK^+^ tubules. Scale bar, 100 µm. (n = 5). (**G** and **H**) SCr and BUN levels in different groups. (n = 5). (**I**) Micrographs show kidney morphology and KIM-1 expression in different groups. Asterisks or arrows indicate injured tubules. Scale bar, 100 µm. (**J** and **K**) Quantitative data are presented. (n = 5). (**L**) Western blot analyses (L) show NGAL, Bax, cleaved caspase3, and c-Fos expression in different groups. (**M - P**) Quantitative data are presented. Numbers (1, 2) indicate each individual animal in a given group. (n = 5). *P < 0.05, **P < 0.01 versus sham controls; †*P* < 0.05, ††*P* < 0.01 versus IRI + limonin; §*P* < 0.05, §§*P* < 0.01 versus IRI + limonin + U0126.

To verify whether limonin protects against ischemic AKI through activating ERK, we concomitantly administrated U0126 with limonin *in vivo*. In brief, limonin was orally administered 3 days before IRI, and U0126 was treated intraperitoneally on the same day with IRI (Figure 9A). At 1 day after AKI, western blot assay showed that limonin induced renal ERK activation in IRI mice, which was eradicated by U0126 treatment (Figure 9, B-D). IHC staining confirmed the changes in ERK phosphorylation occurred mainly in renal tubular cells (Figure 9, E-F). Then, renal functions were evaluated. The levels of SCr and BUN were decreased after limonin treatment in IRI mice (Figure 9, G-H). However, inhibition of ERK by U0126 largely abolished the protective effects of limonin on kidney functions. Accordingly, PAS staining revealed that U0126 also ended the protective effects of limonin on kidney morphology after AKI (Figure 9, I-J). Consistently, IHC staining indicated increased inductions of KIM-1 after blockade of ERK, compared to the IRI+limonin group (Figure 9I and 9K). Similarly, NGAL levels were also increased by combined U0126 treatment, compared to the limonin group (Figure 9, L-M). Furthermore, we examined the effect of U0126 on cell apoptosis and tubular regeneration. U0126 restored the expression of Bax and cleaved caspase3 but inhibited c-Fos expression based on limonin treatment in IRI mice (Figure 9, L and N-P). Together, these data suggested that limonin protects against AKI primarily through activating ERK *in vivo*.

## Discussion

**Figure 10:**
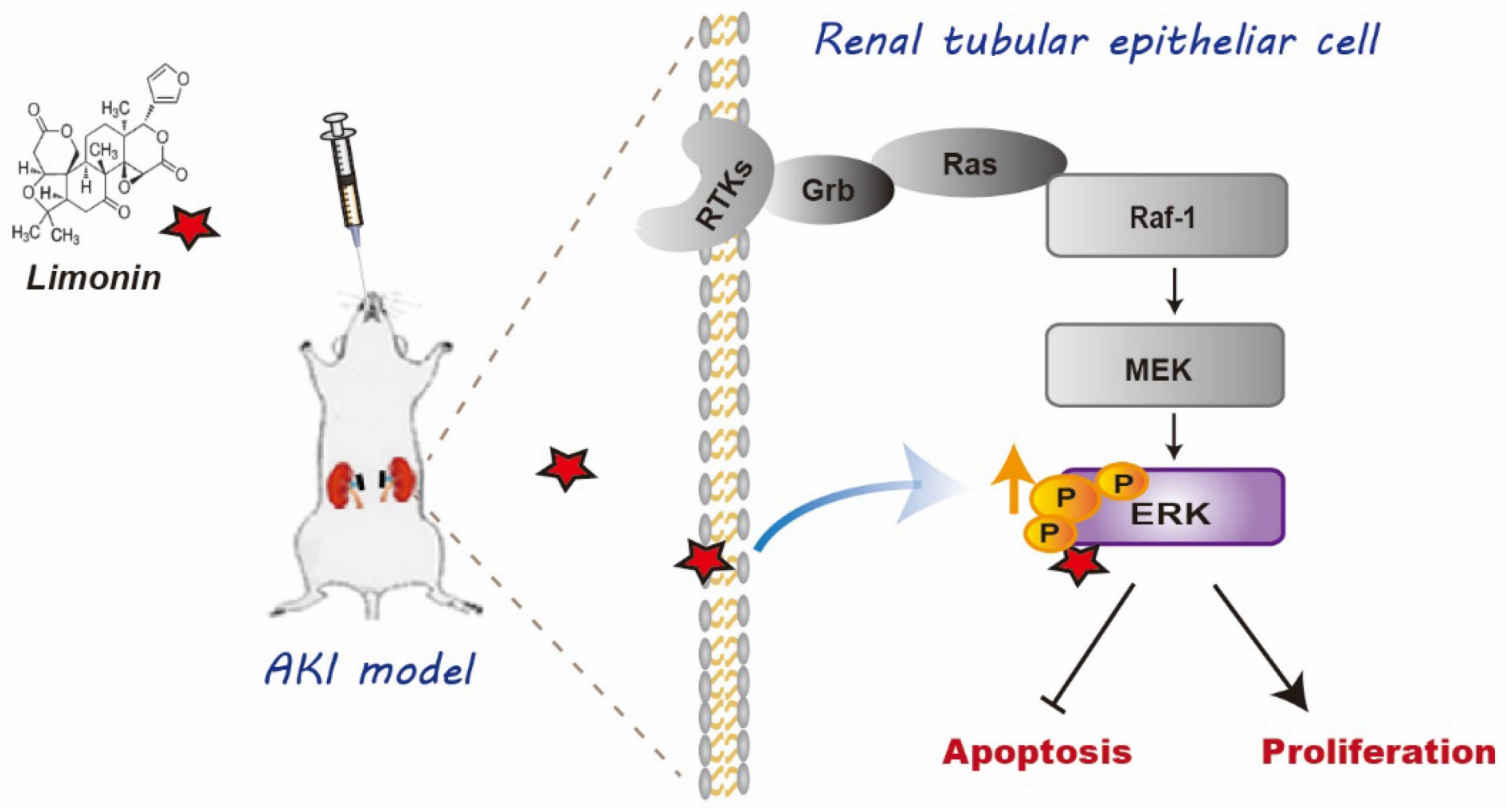
** Diagram shows that Limonin protect AKI.** The schematic graph shows that limonin protects the kidneys against I/R injury through phosphorylating ERK to reduce apoptotic responses and enhance cell proliferation.

In this study, we have provided evidence showing that limonin is a natural agonist of ERK2 that exhibits exceptional renoprotective effects in ischemic AKI. To the best of our knowledge, it is the first time to report that ERK2 is a previously unrecognized target of limonin. As depicted in Figure 10, ERK2 formed hydrogen bonds with limonin in structure, thereby limonin functionally activated ERK2 to protect against ischemic AKI by balancing tubular cell death and proliferation. Our findings provide valuable insights into the cellular and molecular mechanism of how limonin prevents or mitigates AKI in the preclinical setting.

Over the past decades, limonin has attracted increasing attention because of its potential health-promoting capacities such as anti-oxidant, anti-inflammatory, anti-cancer, and organ-protective effects. Our results show that administration of limonin prior to kidney injury fosters improved renal tubular pathology and preserved kidney functions. Accordingly, kidney inflammation and cell apoptosis are ameliorated, and tubular proliferation is enhanced (Figure 1 and 2). The renoprotective effects of limonin has been also validated in cultured tubular cells *in vitro* (Figure 3). Of interest, in various cancer cells, limonin exerts its abilities in pro-apoptosis and anti-proliferation 43-45, which seems utterly distinct from its protective features on repairing tubular cells. There are two possible reasons for this phenomenon. One is cellular effects. Tubular cells are the essential resident components of the kidney tissue, whereas cancer cells are often characterized by unlimited proliferation, invasion, and migration capacities. To some extent, they denote a favorable or a detrimental microenvironment, respectively. Therefore, the cellular responses to limonin might not be equivalent. Another possibility is dose effects. The dosage of limonin applied in treating kidney disease was much lower than it typically used in dealing with cancers.

A novel point of this study is that by exploiting the network pharmacology approach, we identified the protein targets of limonin 46, 47. Multiple targets and pathways are suggested to connect with the renoprotection effects of limonin in AKI (Figure 4). Among those target proteins, ERK2 has been highlighted because tyrosine (Tyr 34), lysine (Lys 52), and cysteine (Cys 164) in ERK2 formed hydrogen bonds with limonin to stabilize the position of limonin in the ERK2 binding pocket. Of note, ERK1 and ERK2 exhibit 80% homology and have similar functions 48. Our western blot assay showed that the change of ERK1 (44KD) and ERK2 (42KD) bands were identical.

Thus, we generally refer to them as ERK when describing our results. However, it should be pointed out that we cannot exclude possibility that other identified top-ranked proteins have the ability to binding with limonin to exert their function in protecting against AKI. Of particular interest, a recent published study reported that limonin also can interact with glycogen synthase kinase-3β (GSK3β) 49, while inhibiting GSK3β activities has been demonstrated is a cost-effective adjuvant strategy for preventing or mitigating AKI 50.

The most exciting finding of our study is that we confirmed that limonin is a unique natural activator of ERK from multiple dimensions. First, both MST and CETSA assays quantitatively approved the solid binding affinity between limonin and ERK2. Second, *in vitro* and* ex vivo* results indicated that limonin-mediated proliferation and anti-apoptosis in tubular cell depend upon ERK phosphorylation (Figure 5 and 6). Frankly speaking, the role of ERK signaling pathway in tissue injury/repair has been obscured for a long period because of the conflicting results in different systems. One theory is that ERK activation protects the ischemic kidney by reducing cell apoptosis 51, 52, whereas several studies reported that suppressing ERK signaling improved cell survival after IRI in the kidney 42, 53-56. As we know, the ERK signaling pathway is typically activated by growth factor receptors and vasoactive molecules. Therefore, whether ERK plays a positive or negative role in AKI largely depends on its surrounding microenvironment. In this study, limonin activated ERK and exerted a positive response in protecting against AKI. Besides activating ERK, limonin possesses potent anti-inflammation capacity such as represses macrophage activation, we consequently speculated that limonin has already built a favorable microenvironment for ERK activation, creating a “win-win” situation after ischemic AKI. On the other hand, the injury model difference might be another reason for the opposite role of ERK in AKI. In the current study, we employed an ischemic AKI model. The pathologic feature of the kidney IRI model is the injury spots initiated from the juxtamedullary nephrons because this area is much more sensitive to ischemia and hypoxia. Subsequently, these pathological changes may cause disturbance of ion exchanges and distinct patterns of activated or inactivated growth factors and enzymes if compared with toxin-induced AKI models. The integration of signaling pathways under different experimental conditions is certainly vary. These factors together could lead ERK to play diverse roles after AKI. Our findings indicate the beneficial role of kidney ERK phosphorylation in ischemic AKI. Most importantly, the connection between limonin and ERK signaling is fully supported by the *in vivo* evidence. Inhibition of ERK signaling by U0126 significantly abolishes limonin-mediated renoprotection against AKI (Figure 9). Based on these findings, we report that limonin serves as an ERK agonist to promote the recovery of ischemic AKI.

Earlier studies have shown that limonin has poor bioavailability due to its low water solubility and permeability. As a result, it takes a long time to reach peak blood concentration 57-59. In our study, we made some optimizations to enhance limonin's bioavailability. First, HS15 60, a novel solubilizer with high solubilizing capacity and excellent safety, was used to prepare the limonin solution. Second, limonin was administered once daily at 80 mg/kg for four consecutive days to maintain higher serum drug concentration. Further structural optimization is required to obtain more potent limonin derivatives. In addition, limonin has reasonably good safety, which has already been proven in Phase I clinical trials 61. Our preclinical studies in mice also provided evidence for the clinical evaluation of AKI patients. A limitation of our study is that we only utilized a single ischemic AKI model. Thus, multiple AKI models caused by different etiologies are needed to validate our findings.

In conclusion, we herein state that limonin is an ERK2 agonist that possesses the capacity to protect against ischemic AKI. Our *in vivo*, *in vitro*, and *ex vivo* results clearly illustrated that limonin reduces cell death and promotes tubule repair and regeneration through activating ERK. Although further exploration is needed, our study shed light on developing effective natural therapeutic agents to enhance our abilities in tackling the increasing medical burden of AKI globally.

## Supplementary Material

Supplementary figures and tables.

## Abbreviations

AKI: acute kidney injury
ALT: Alanine aminotransferase
AST: Aspartate aminotransferase
BUN: blood urea nitrogen
CETSA: cellular thermal shift assay
CKD: chronic kidney disease
EdU: 5-ethynyl-2′-deoxyuridin
ERK2: Extracellular signal-regulated kinase 2
ESRD: end stage renal disease
H/R: hypoxia/reoxygenation
IRI: ischemia-reperfusion injury
KEGG: Kyoto Encyclopedia of Gene and Genomes
MAPK1: Mitogen-activated protein kinase 1
MEK: Mitogen-activated protein kinase kinase
MST: Microscale thermophoresis
PAS: Periodic acid-Schiff
SCr: serum creatinine
TECs: tubular epithelial cells
TUNEL: Terminal deoxynucleotidyl transferase-mediated deoxyuridine triphosphate nick end labeling
