# Supplementary material for: Limonin, a natural ERK2 agonist, protects against ischemic acute kidney injury
Source: Int J Biol Sci. 2023 May 29;19(9):2860–78. doi: 10.7150/ijbs.82417 (PMC10266085; doi:10.7150/ijbs.82417)
Supplement: Supplementary file 1 — Supplementary figures and tables. [file ijbsv19p2860s1.pdf]

Supplementary Materials

1. Supplementary Tables (Table S1 to S6)

2. Supplementary Figures (Figure S1 to S4)

1. Supplementary tables

Table S1. The analysis of blood biochemistry in mice treated with or without limonin.

Biochemical index	Control (HS15)	Limonin (80 mg/kg)
**SCr**	0.19±0.02	0.18±0.02
**BUN**	27.72±1.29	24.12±1.54
**ALT**	34.5±6.66	26.1±3.82
**ALB**	23.46±0.49	24.96±0.84
**CHOL**	2.33±0.19	2.6±0.14
**TG**	1.32±0.12	1.11±0.12
**HDL**	1.43±0.04	1.7±0.08
**LDL**	0.49±0.09	0.44±0.04

Table S2. Model enrichment of limonin using PharmMapper.

Number	Pharma model	Target protein	Fit score
1	1O6U	SEC14-like protein 2	5
2	1J78	Vitamin D-binding protein	4
3	2FKY	Kinesin-like protein KIF11	4
4	1N83	Nuclear receptor ROR-alpha	4
5	1L6L	Apolipoprotein A-II	3
6	1OJ9	Amine oxidase [flavin-containing] B	3
7	1REU	Bone morphogenetic protein 2	3
8	1J96	Aldo-keto reductase family 1 member C2	3
9	1P49	Steryl-sulfatase	3
10	1PME	Mitogen-activated protein kinase 1	3

Table S3. Topological characteristics of the crucial targets from the network.

Number	Target protein	Degree	Closeness centrality	Betweenness centrality
1	SRC	34	0.1230629	2540.1274
2	MAPK1	33	0.12295082	2554.1855
3	PIK3R1	33	0.12032086	1077.3319
4	AKT1	30	0.12032086	2404.1838
5	GRB2	28	0.11883803	422.3124
6	PTPN11	27	0.11862917	311.3821
7	HSP90AA1	26	0.12075134	1694.7158
8	MAPK14	23	0.119152695	1452.9944
9	ESR1	20	0.11904762	1132.8658
10	EGFR	20	0.11769834	399.86707

Table S4. Tm50 value of ERK protein and α-Tubulin.

Molecular	Control (℃)	Limonin (℃)	ΔTm50 (℃)
**ERK**	53.05 ± 1.471	59.37 ± 1.379	-6.315 ± 2.016
**α-Tubulin**	59.57 ± 1.564	58.89 ± 0.9317	-0.676 ± 1.82

Table S5. The websites used in this study.

**Database**	**URL**
Bioinformatics	http: //www.bioinformatics.com.cn/
DAVID	http: //david.ncifcrf.gov/
GeneCards	https://www.genecards.org/
PharmMapper Server	http: //lilab-ecust.edu.cn/pharmmapper/
Protein Data Bank	http: //www.rcsb.org/
Pubchem	http: //pubchem.ncbi.nlm.nih.gov/
Uniprot	http: //www.uniprot.org/
STRING	http: //string-db.org/

Table S6. The sources of antibodies used in this study.

Antibodies	Catalogue number	Company
**Primary antibodies**		
anti-pERK1/2	#9101	Cell Signaling Technology
anti-total ERK	#9102	Cell Signaling Technology
anti-pMEK	#9154	Cell Signaling Technology
anti-total MEK	#4694	Cell Signaling Technology
anti-PARP	#9542	Cell Signaling Technology
anti-Fas	#8023	Cell Signaling Technology
anti-FADD	#2782	Cell Signaling Technology
anti-Bcl-2	#15071	Cell Signaling Technology
anti-Caspase3	#9662	Cell Signaling Technology
anti-Caspase7	#8438	Cell Signaling Technology
anti-Kim-1	AF1817	R & D Systems
anti-NGAL	ab63929	Abcam
anti-TNF-α	ab9739	Abcam
anti-IL-6	ab7737	Abcam
anti-CD68	ab955	Abcam
anti-Laminin	ab11575	Abcam
anti-p53	sc-126	Santa Cruz Biotechnology
anti-FasL	sc-33716	Santa Cruz Biotechnology
anti-PCNA	#2586	Santa Cruz Biotechnolog
anti-FADD	sc-271748	Santa Cruz Biotechnology
anti-Bax	sc-7480	Santa Cruz Biotechnology
anti-Cyclin D1	sc-753	Santa Cruz Biotechnology
anti-Survivin	PB0377	Boster Biological Technology
anti-c-Fos	BA0207-2	Boster Biological Technology
anti-α-Tubulin	RM2007	Ray Antibody Biotech
**Secondary antibodies**		
Goat anti-Mouse	BA1050	Boster Biological Technology
Goat anti-Rabbit	BA1054	Boster Biological Technology
Rabbit anti-Goat	BA1060	Boster Biological Technology
Donkey anti-Mouse	715-065-150	Jackson ImmunoResearch
Donkey anti-Rabbit	711-065-152	Jackson ImmunoResearch
Donkey anti-Goat	705-065-003	Jackson ImmunoResearch
Cy2-conjugated Donkey anti-Mouse IgG	715-225-150	Jackson ImmunoResearch
Cy3-conjugated Donkey anti-Rabbit IgG	711-165-152	Jackson ImmunoResearch

2. Supplementary Figures

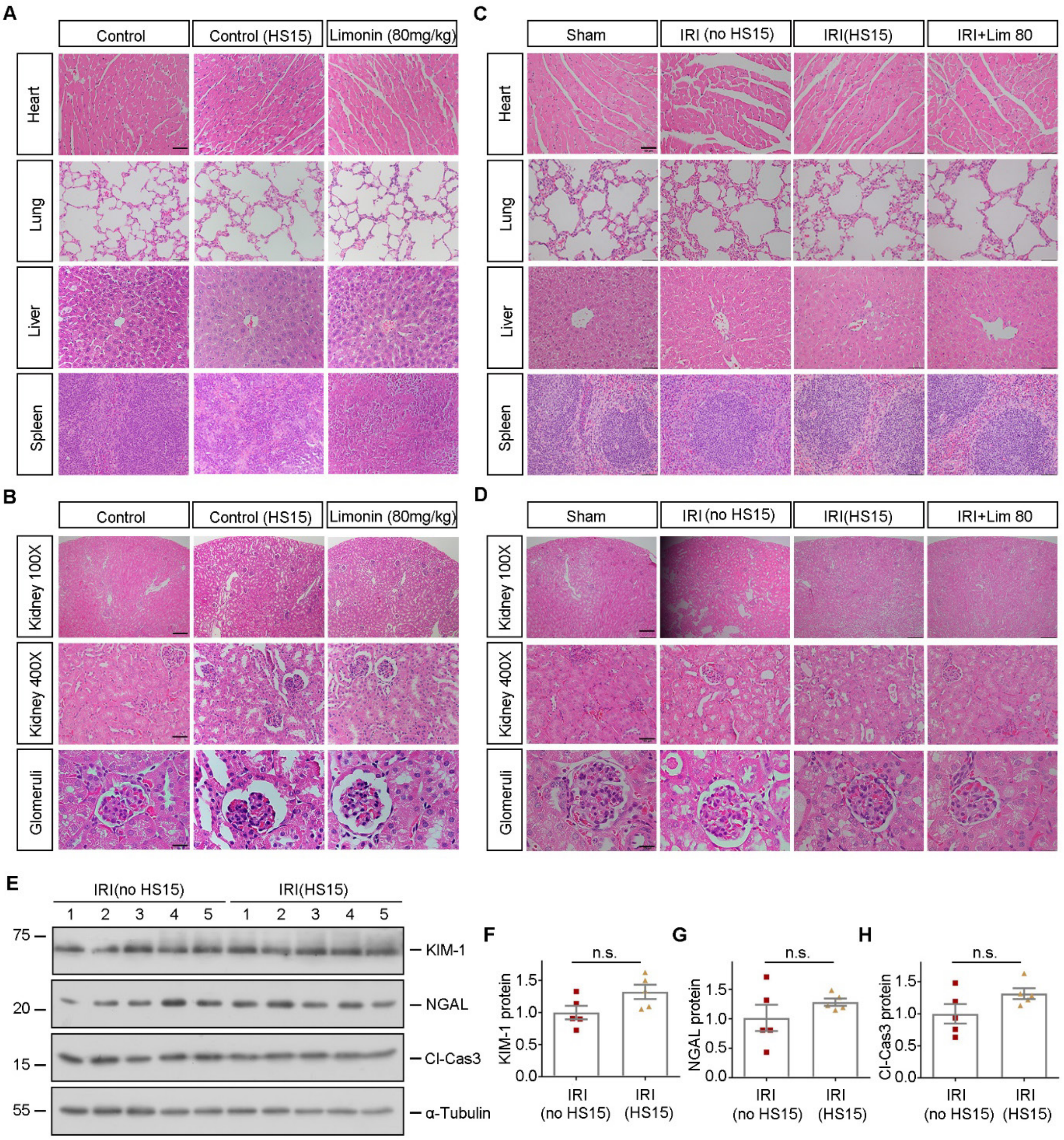

Figure S1. Limonin does not cause systemic toxicities *in vivo*.

Representative micrographs of HE staining of heart, lung, liver, spleen (**A**), and kidney (**B**) in normal mice. (**A**) Scale bar, 50 μm. (**B**) Scale bar, 200 μm (upper panels); 50 μm (middle panels); 20 μm (lower panels). Representative micrographs of HE staining of heart, lung, liver, spleen (**C**) and kidney (**D**) in IRI-AKI mice. (**C**) Scale bar, 50 μm. (**D**) Scale bar, 200 μm (upper panels); 50 μm (middle panels); 20 μm (lower panels) in IRI-AKI mice. (**E**) Western blot analyses show KIM-1, NGAL, cleaved-caspase3 expression in IRI and IRI+HS15 groups. Quantitative data (**F-H**) are presented. Numbers (1-5) indicate each individual animal in a given group. (n = 5).

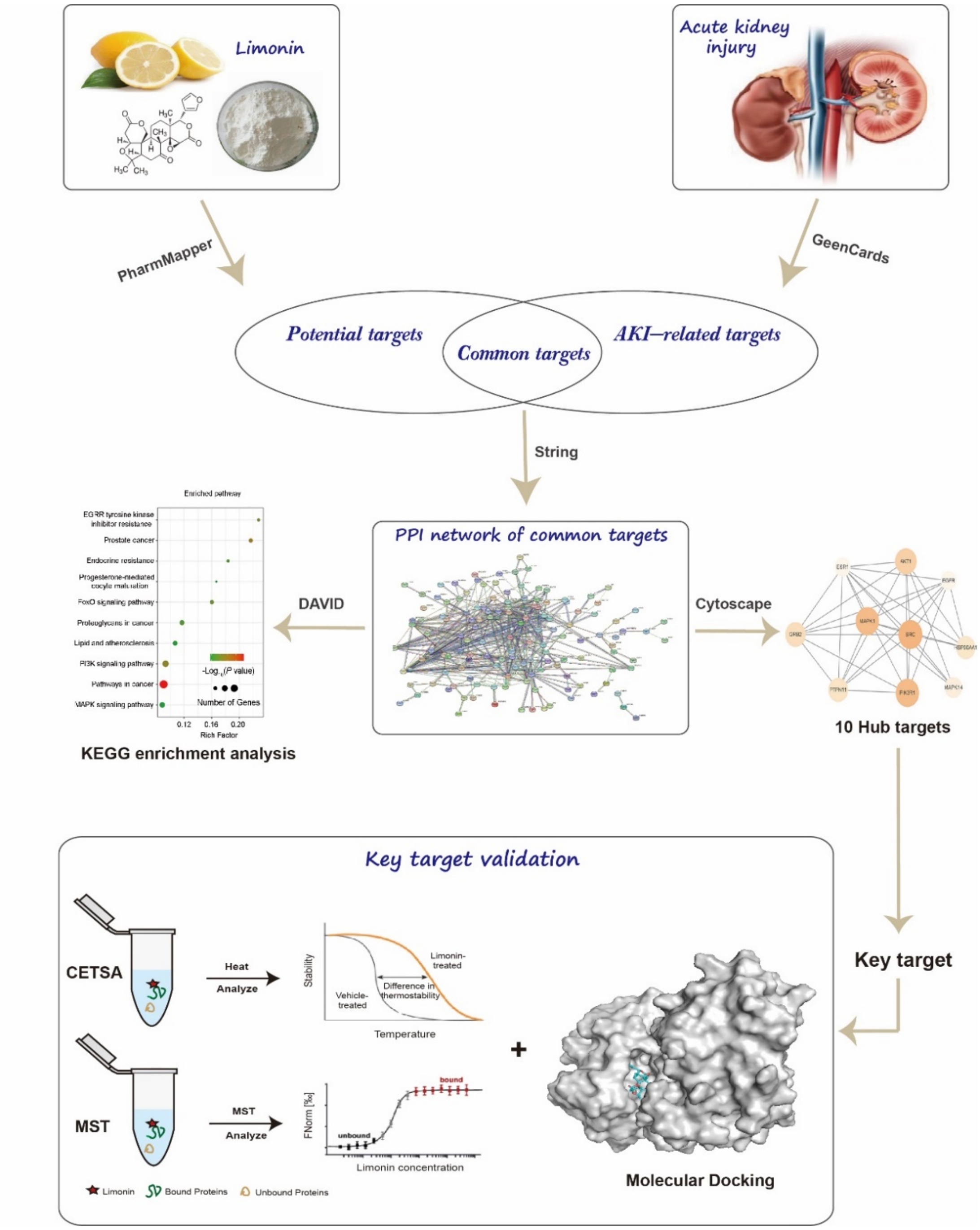

Figure S2. The flow chart for researching the potential targets of limonin in treating AKI.

The potential targets of limonin were obtained through Pharmmapper. The AKI-related targets were downloaded from GeenCards database, and the common targets were identified by intersecting limonin targets and AKI-related targets. PPI network of common targets was performed. KEGG enrichment analysis and Cytoscape were used to select hub target. Finally, the interaction of key target and limonin was validated by molecular docking, cellular thermal shift assay and MicroScale Thermophoresis.

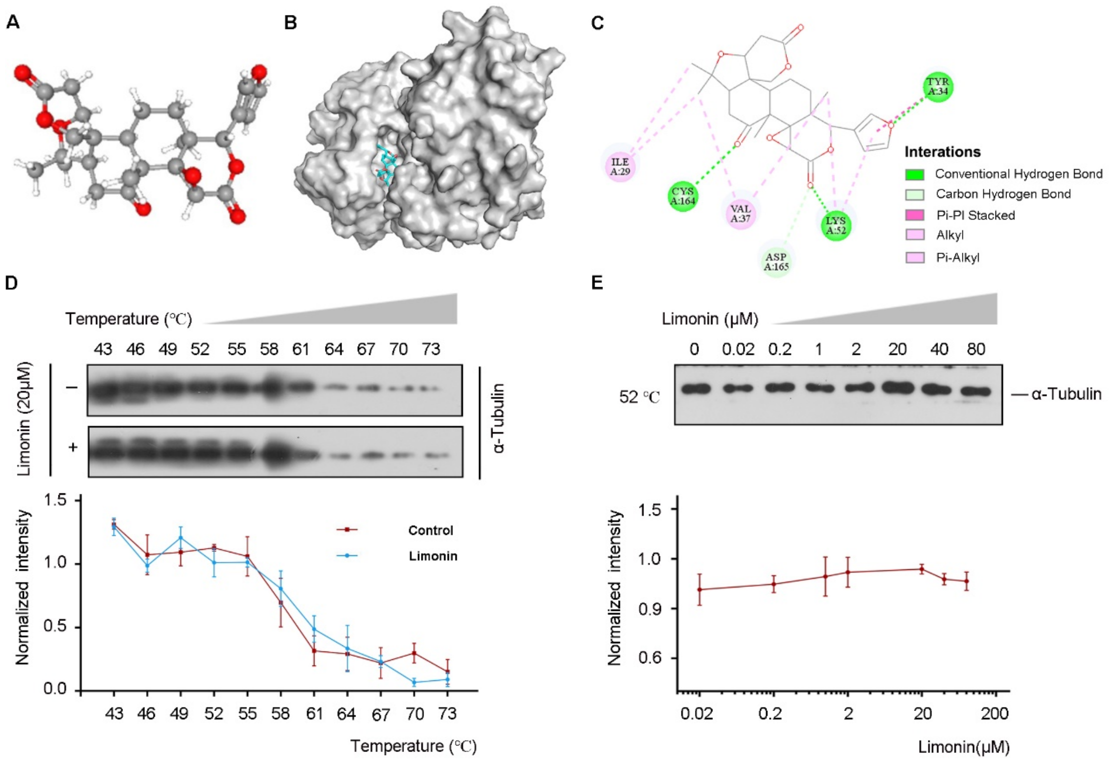

Figure S3. The thermal shift assay of a control protein upon limonin treatment.

(**A**) 3-D structure of limonin. (**B, C**) Potential binding sites of limonin and ERK2. (**D, E**) Limonin treatment didn't affect the thermal stability of α-tubulin in cell lysates as measured by the temperature-dependent cellular thermal shift assay (**D**) or the concentration-dependent cellular thermal shift assay at 52°C (**E**). n = 3 biologically independent experiments.

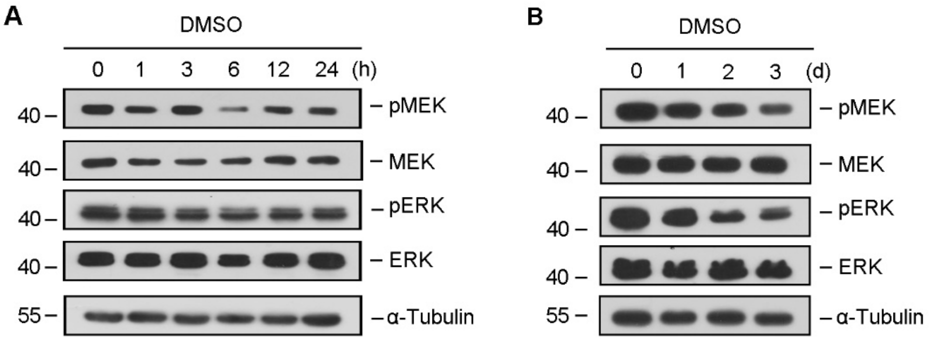

Figure S4. The changes of ERK and MEK in HK-2 cells after treated with solvent.
(**A**, **B**) HK-2 cells were incubated with solvent (DMSO) for various periods of time as indicated. Cell lysates were subjected to immunoblots with specific antibodies. In short- (**A**) or long-term (**B**) experiments, representative Western blots show pMEK and pERK changes.
